# Differences of the Morphology of Subaxial Cervical Spine Endplates between Chinese and White Men and Women

**DOI:** 10.1155/2018/2854175

**Published:** 2018-02-20

**Authors:** Qi Yao, Peng Yin, Kamran Khan, Tsung-Yuan Tsai, Jing-Sheng Li, Yong Hai, Peifu Tang, Guoan Li

**Affiliations:** ^1^Department of Orthopedics, Chinese PLA General Hospital, No. 28 Fuxin Road, Beijing 100853, China; ^2^Department of Orthopedics, Beijing Shijitan Hospital, Capital Medical University, Beijing 100037, China; ^3^Department of Orthopedics, Beijing Chaoyang Hospital, Capital Medical University, Beijing 100020, China; ^4^Bioengineering Laboratory, Department of Orthopedic Surgery, Massachusetts General Hospital and Harvard Medical School, Boston, MA, USA

## Abstract

*Objective. *The aim of this comparative anatomical study was to specifically investigate endplate morphology differences between Chinese and White men and women.* Materials and Methods. *Three-dimensional cervical endplate models were constructed using computed tomography imaging of 41 healthy Chinese and 24 White subjects. The morphologic measurements of cervical endplate included linear parameters (EPWu: upper endplate width; EPDu: upper endplate depth; EPWl: lower endplate width; and EPDl: lower endplate depth) and area parameters with a digital measuring system.* Results. *All linear parameters showed a constant increase from C3 to C7 except for EPDl in both the Chinese and the White subjects. An increase trend was observed on area parameters in both Chinese and White subjects. The ratio of EPWl/EPDl was smaller in Chinese females than in White females at C3, C4, and C6 levels (*P* < 0.05). The ratio of EPWl/EPDl was significantly different between the Chinese and White men at C4-5 levels (*P* < 0.05).* Conclusions. *Our data indicates that the morphology of subaxial cervical spine endplates between Chinese and White men and women is different in most of the linear and area parameters. This information could provide guidelines for the design of CDA implants and the improvement of surgical techniques.

## 1. Introduction

Lately, there has been an increase in the use of artificial cervical disc arthroplasty (ACDA) for degenerative conditions as an alternative to spinal fusion [1]. However, implant dislocation and subsidence are often mentioned as the main postoperative complications associated with ACDA [2–5]. The success of this motion preserving procedure relies upon proper sizing and positioning of the implants at the cervical endplates. Therefore, accurate knowledge of the morphology of the cervical vertebral endplate is vital for the development of spinal implants and surgical instruments to replace the diseased disc.

Limited data has been reported on the morphology of cervical endplate. Previous investigators have sought to analyze the anatomy of the cervical vertebral bodies and endplates based on measurements derived from plain radiographs, cadaveric specimens, and computed tomography (CT) scans [6–9]. Panjabi et al. [6] measured cervical vertebrae specimens and reported endplate parameters such as width and depth, area, and inclination in White subjects. Tan et al. [7] reported the endplate parameters in Singaporean and Korean subjects separately. Cunningham et al. performed a detailed study on the PCM endplate geometry, such as surface area and height [9]. Recently, CT scans have provided accurate 3D modeling capabilities to accurately study the cervical morphology [8]. Yet, no study has quantitatively investigated the overall parameters of the cervical endplate in living subjects. A literature review further indicated that there has not been any studies specifically investigating endplate morphology differences between Chinese and White men and women.

Cervical disc arthroplasty does not take racial differences into consideration and most of the prosthesis are designed according to anthropometric data obtained from White patients. Furthermore, with the increasing use of CDA in China [10, 11], it is absolutely essential to understand the differences between the endplate morphology of the Chinese and White populations. The aim of the present study was to investigate the anthropometry of the Chinese and White subjects' endplate using 3-dimensional cervical endplate models. We hypothesize that there are distinct differences in size and shape between the Chinese subjects' cervical endplates and the White subjects' cervical endplates.

## 2. Materials and Methods

### 2.1. Subject Demographics

41 Chinese subjects (21 females and 20 males) and 24 White subjects (12 females and 12 males) were recruited for our study. The subjects were evaluated for no history of neck pain, cervical spinal disorders, and anatomical anomalies using clinical and radiographic examinations. The study was approved by our Institutional Review Board, and written consent was obtained from all subjects. The mean age and height of the subjects are listed in Table 1.

### 2.2. Creation of 3-Dimensional Endplate Models

For the Chinese subjects, a CT scan of the endplate was obtained using a helical CT scanner (Siemens, Germany). The scanning procedure was performed to acquire 0.625 mm axial CT slices with a resolution of 512 × 512 pixels. For the White subjects, the scanning procedure was performed to acquire 0.625 mm axial CT slices in a General Electric Light Speed Pro16 CT scanner and resolution of 512 × 512 pixels were obtained.

**Figure 1:**
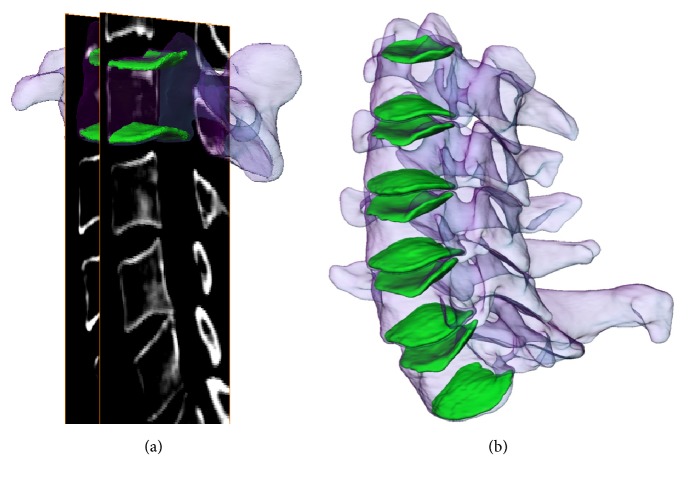
(a) Digitization of the endplates from sagittal CT images. (b) Reconstruction of the 3D models of upper and lower endplates from C3 to C7.

The images of the upper and lower bony endplates from C3 to C7 (total of 650 endplates) were then 3D reconstructed using a solid modeling software (Rhinoceros Robert McNeel & Associates, Seattle, Washington). Window settings for cortical digitization had a width of 100 Hounsfield units (HU) and center at 500 HU (Figure 1(a)). Our previous validation study indicated that similar bony models can be accurately reconstructed using CT images [12].

### 2.3. Measurement of the Endplates

**Figure 2:**
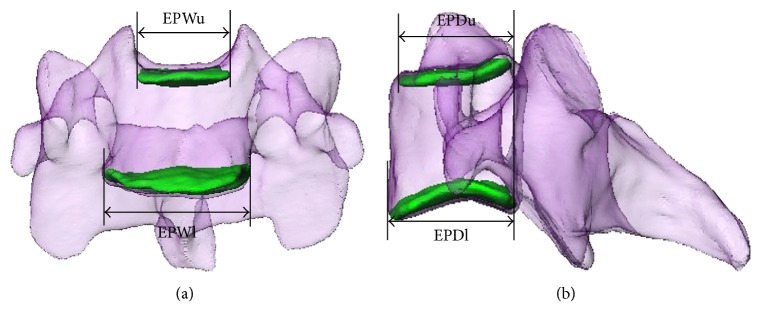
Two orthogonal views (coronal plane (a) and sagittal plane (b) of a cervical vertebra are shown. EPWu: upper endplate width; EPWl: lower endplate width; EPDu: upper endplate depth; EPDl: lower endplate depth.

To analyze the 3D surface anatomy of the middle and lower cervical vertebral endplates, an automatic algorithm was programmed to systematically calculate the endplate from the obtained 3D models. To do this, first a characteristic transverse plane for each endplate was generated through fitting based on least distances of the points on the endplate to the plane (Figure 2). Therefore, the characteristic plane followed the overall endplate direction. Then the shape of the endplate was obtained by projecting the endplate onto the transverse plane, and endplate area was calculated. In addition, the AP direction of each vertebra was determined by connecting the middle of the vertebra body and the tip of the spinous process. The left-right (LR) direction was kept perpendicular to the AP direction in the transverse plane. Endplate width and depth were measured along the LR and AP directions, respectively (Figure 2). (1) Linear parameters include EPWu (upper endplate width), EPDu (upper endplate depth), EPWl (lower endplate width), and EPDl (lower endplate depth); (2) area parameters include EPAu (upper endplate area) and EPAl (lower endplate area).

### 2.4. Statistical Analysis

A Student's *t*-test was performed to determine if the morphological parameters were statistically different between the races in the same sex (SAS Institute Inc., Cary, NC, USA, version 9.1.3). A *P* value of less than 0.05 was considered statistically significant.

## 3. Results

### 3.1. Chinese and White

The article described the results from 650 middle and lower cervical (C3–C7) endplates. The morphological measurements of the endplate were summarized by race (Table 2). In general, all linear parameters were observed to monotonically increase from C3 to C7 except EPDl in both the Chinese and White subjects. There is a slight decrease for EPDl from C6 to C7. From C3 to C5, the average width of upper and lower cervical endplates was significantly smaller in Chinese than in White (*P* < 0.05) subjects. The average EPWu at C6 level was significantly smaller in Chinese than in White (*P* = 0.03) subjects, but there was no significant difference in EPWl at C6 level between the Chinese and White (*P* = 0.39) subjects. The average depth of upper and lower endplates between C5 and C7 was significantly smaller in Chinese than in White (*P* < 0.05) subjects. There was no significant difference in the ratios of EPWu/EPDu and EPWl/EPDl at C3/C5–7 levels between the Chinese and White subjects (*P* > 0.05). At C4 level, the ratio of EPWl/EPDl in Chinese was smaller than in White (*P* = 0.03) subjects, but there was no significant difference in the ratio of EPWu/EPDu (*P* = 0.33).

All area parameters were observed to constantly increase from C3 to C7 in both the Chinese and White subjects. The average areas of cervical endplates at different levels ranged from 169.9 mm^2^ to 281.5 mm^2^ and 204.5 mm^2^ to 316.8 mm^2^ for Chinese and White subjects, respectively (Table 3). Measurement of area showed that the Chinese subjects' area was generally smaller than that of White subjects' area except for C7 upper endplate (*P* < 0.05).

### 3.2. Chinese and White Females

**Figure 3:**
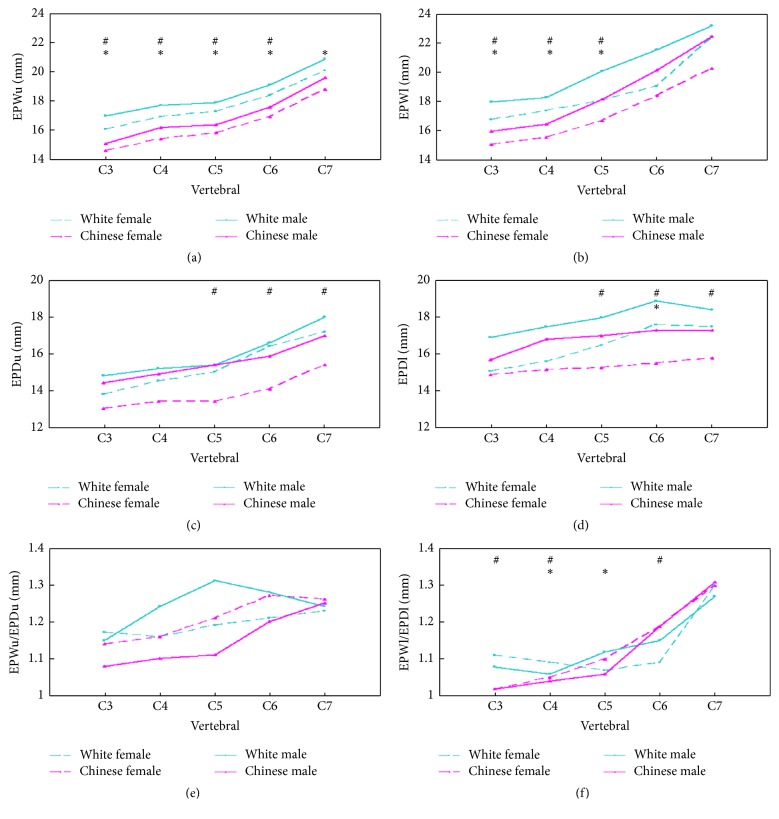
(a), (b), (c), and (d) show the linear dimensions of the upper and lower endplates. (e) and (f) show the ratio of endplate width to depth for the upper and lower endplates. #: differed significantly between the Chinese females and White females; *∗*: differed significantly between the males and White males.

The average width of cervical endplates at different levels ranged from 14.6 mm to 20.3 mm and from 16.1 mm to 22.4 mm in Chinese and White females, respectively. The average depth of cervical endplates at different levels ranged from 13.0 mm to 15.8 mm and from 13.8 mm to 17.5 mm for Chinese and White females, respectively (Table 4). The EPWu of Chinese females was significantly smaller than that of White females from C3 to C6 (*P* < 0.05) (Figure 3(a)). The EPWl of Chinese females was significantly smaller than that of White females from C3 to C5 (*P* < 0.05) (Figure 3(b)). The EPDu and EPDl were significantly smaller in Chinese females than in White females from C5 to C7 (*P* < 0.05) (Figures 3(c) and 3(d)). The difference in EPWu/EPDu ratios was not statistically significant from C3 to C7 (*P* > 0.05) (Figure 3(e)). A statistically significant difference was noted between EPWl/EPDl ratios at C3, C4, and C6 levels (*P* < 0.05) (Figure 3(f)).

The average areas of cervical endplates at different levels ranged from 169.9 mm^2^ to 281.5 mm^2^ and from 204.5 mm^2^ to 316.8 mm^2^ for Chinese females and White females, respectively. Measurement of area showed that the EPAu of Chinese females was generally smaller than that of White females from C3 to C6 (*P* < 0.05). The EPAl showed no difference between the 2 groups (*P* > 0.05) (Table 5).

### 3.3. Chinese and White Males (Table 4)

The average width of cervical endplates at different levels ranged from 15.1 mm to 22.5 mm and from 17.0 mm to 23.2 mm for Chinese and White males, respectively. The average depth of cervical endplates at different levels ranged from 14.4 mm to 17.3 mm and from 14.8 mm to 18.4 mm for Chinese and White males, respectively (Table 4). The EPWu of Chinese males was significantly smaller than that of White males from C3 to C7 (*P* < 0.05) (Figure 3(a)). The EPWl of Chinese males was significantly smaller than that of White males from C3 to C5 (*P* < 0.05) (Figure 3(b)). The difference in EPDu was not statistically significant from C3 to C7 (*P* > 0.05) (Figure 3(c)). The difference in EPDl was not statistically significant except for C6 (Figure 3(d)) (*P* > 0.05). The EPWu/EPDu ratio was not statistically significant from C3 to C7 (*P* > 0.05) (Figure 3(e)). A statistically significant difference was noted between EPWl/EPDl ratios at C4 and C5 levels. (Figure 3(f)).

The average areas of cervical endplates at different levels ranged from 171.2 mm^2^ to 290.7 mm^2^ and 216.1 mm^2^ to 335.2 mm^2^ for Chinese and White males, respectively. Measurement of area showed that the EPAu of Chinese males was generally smaller than that of White males from C3 to C6 (*P* < 0.05). The EPAl showed a significant difference except for C5 between Chinese and White males (*P* < 0.05) (Table 5).

## 4. Discussion

The present study showed that the subaxial cervical spine endplates of Chinese males and females were smaller than those of their White counterparts in most linear and area parameters. In Chinese females, the EPWu and EPWl were smaller than in White females from C3 to C5; and the EPDu and EPDl were smaller than in White females from C5 to C7. The ratio of EPWl/EPDl was smaller in Chinese females than in White females at C3, C4, and C6 levels. The EPAu of Chinese females was smaller than that of White females from C3 to C6. In Chinese males, the EPWu and EPWl were significantly smaller than in White males from C3 to C5, and the only significant difference was observed at C6 in EPDl. The ratio of EPWl/EPDl was significantly different between the Chinese and White men at C4-5 levels. The EPAu and EPAl of Chinese males were smaller than those of White males at C3, C4, and C6 levels. These results proved the hypothesis that there are distinct differences in size and shape between the Chinese and White subaxial cervical spine endplates.

Many studies have reported on the measurement of cervical spine endplates. Panjabi et al. studied endplate morphology from 12 cadaveric cervical spine specimens using the morphometer, a device used to define points on the measuring surface. These results agree quite closely with our measurements of the endplates of living White subjects of the EPWu, EPDu, EPWl, and EPDl. Tan et al. studied 10 cadavers of Chinese Singaporean subjects with a digitizer, following the measurements proposed by Panjabi, and compared the results to the White population. They found similar trends with our study and noticed that the dimensions of the Singaporeans were smaller than the Caucasians. Kim et al. utilized CT scans to measure the endplate morphology of 57 Korean cadavers and compared them with the previous studies. They found deeper but narrower endplates in the Koreans than in the Caucasians and overall larger dimensions than in the Singaporeans, but still smaller than in the Whites. Interestingly, they reported smaller endplate width than depth from C3 to C6, which is different from the results of the current study of the Chinese, the study of the Caucasians reported by Panjabi, and that of the Singaporeans reported by Tan. Comparing our data to these previous studies, the values for all the selected parameters in living Chinese cervical endplates were considerably larger than those reported for Singaporean subjects by 1 to 2 mm. The EPWu values in living Chinese cervical endplate were considerably larger than those reported for Korean subjects by 1 to 2 mm. The EPDu, EPWl, and EPDl values in living Chinese cervical endplate were considerably smaller than those reported for Korean subjects by 1 to 3 mm. The width to depth ratio of the endplate increased from C3 to C7 and similar Singaporean subjects.

The anatomical results from this study on the cervical endplates could provide guidelines for implant sizing and prosthesis design. Multiple studies have reported from 1.2% to 11% reoperation rates after CDA at 2-year followup [13–18]. Currently, the most prevalent types of failure found on followup imaging are subtle implant subsidence, migration, and loosening, although the majority of them are asymptomatic in the short term [4]. These commonly result from poor implant sizing, positioning, and shape mismatch. A small implant may not have enough recover rates and may result in high stress to cause subsidence. An oversized implant may have problems of protrusion and thus may compress the nerves and soft tissues, which can further cause clinical issues and diseases. Shape mismatch between the implant and the endplate is the most common reason for migration and loosening. Furthermore, our results regarding the dimensions and shape of males and females, at different vertebral levels and of inferior and superior disc endplates, may provide guidance to help reduce the complications related to endplate-implant interface after CDA. Currently, there is no consensus on the optimized cervical prosthesis shape to sit on the endplate. For example, the ProDisc-C (DePuy Synthes) has a rectangular shape with rounded corners, the Prestige disc (Medtronic) has a triangular shape, and the Bryan disc has a circular shape (Medtronic) [19]. The anatomical endplate shapes analyzed in this study may provide better quantitative understanding for anatomical implant design and help decrease postoperation complications.

Morphometric evaluation of the vertebral is not a new subject in study of the cervical spine. With the numerous technologic advancements in CT over the last decade, the CT based methods of measurement is now able to precisely evaluate the cervical vertebral dimensions. Main methods of the previous studies used digitizers or CT scans on cadavers. However, it should be stated that our data were measured from the living subjects. Despite the little influence on morphological measurements, in vivo methods have potential advantages for subsequential biomechanical studies, such as kinematic/kinetic modeling, finite element analysis, and bone quality assessment [20]. In addition, cadaver spines can be difficult and costly to obtain and investigate in a large scale.

The current study had several limitations. One of the prominent limitations of the present study is the relatively small size of the subjects. This study included data from 65 healthy subjects. If a larger size was studied then other significant differences may also have been revealed. In addition, all the Chinese subjects recruited in the present study perhaps only represented the subgroup population of Chinese. However, the magnitude of the difference likely remains small since other studies have also utilized a small number of subjects with reliable results. We are continuing to collect more subjects for each group. In future studies, we will also examine the ACD (artificial cervical disc) mismatch data for Chinese patients, and these data can be useful for designing systems. The study has been presented in the 8th International Congress of Chinese Orthopaedic Association [21].

To conclude, the present study used an in vivo CT approach to quantitatively investigate the dimensions and shape of cervical endplates. Significant differences were observed between Chinese and White men and women in most linear and area parameters at various vertebral levels. These statistically significant results provide baseline information for design of CDA implants and surgical techniques. These data should be used to create precise and accurate new disc prosthesis so complications arising from cervical disc arthroplasty could be reduced and patients' health could be restored faster.

## Figures and Tables

**Table 1:** The mean age and height of the subjects.

Characteristic	Female			Male		
	Chinese	White	*P* value	Chinese	White	*P* value
Age (years)	28.2 ± 15.1	29.4 ± 12.3	*P* = 0.81	27.2 ± 12.1	28.9 ± 11.4	*P* = 0.69
Height (cm)	161.2 ± 5.6	166.4 ± 6.2	*P* = 0.02	173.2 ± 4.6	178.4 ± 5.3	*P* = 0.006

**Table 2:** Dimensions of linear parameters categorized by race.

	Race	C3	C4	C5	C6	C7
EPWu (mm)	Chinese	14.6 ± 1.8	15.8 ± 1.7	16 ± 2.1	17.2 ± 2.3	19.1 ± 2.5
	White	16.6 ± 2.4	17.3 ± 2.4	17.6 ± 2.8	18.7 ± 2.5	19.5 ± 2.2
	*P* value	0.03	0.02	0.02	0.03	0.36

EPDu (mm)	Chinese	13.6 ± 1.8	14.2 ± 2.1	14.4 ± 2.2	15 ± 2	16.2 ± 2
	White	14.5 ± 1.8	14.9 ± 1.8	15.2 ± 1.8	16.5 ± 2	17.7 ± 2.2
	*P* value	0.38	0.65	0.04	0.03	0.03

EPWl (mm)	Chinese	16 ± 2	16 ± 2.1	17.4 ± 2.2	19.3 ± 2.8	21.4 ± 3.3
	White	17.5 ± 2.7	17.9 ± 2.7	19.4 ± 1.9	20.6 ± 2.4	22.9 ± 2.8
	*P* value	0.01	0.03	0.04	0.39	0.16

EPDl (mm)	Chinese	15.3 ± 0.18	15.9 ± 2	16.4 ± 2.3	16.5 ± 2	16.3 ± 2.3
	White	16.1 ± 2.1	16.2 ± 2.2	16.9 ± 2	18.4 ± 1.7	18 ± 1.9
	*P* value	0.56	0.43	0.03	0.01	0.04

EPWu/EPDu	Chinese	1.09 ± 0.14	1.12 ± 0.12	1.12 ± 0.11	1.16 ± 0.15	1.19 ± 0.14
	White	1.16 ± 0.12	1.18 ± 0.19	1.19 ± 0.26	1.16 ± 0.24	1.18 ± 0.17
	*P* value	0.66	0.33	0.79	0.93	0.82

EPWl/EPDl	Chinese	1.01 ± 0.13	1.02 ± 0.13	1.06 ± 0.11	1.19 ± 0.14	1.31 ± 0.22
	White	1.09 ± 0.17	1.1 ± 0.12	1.13 ± 0.14	1.12 ± 0.11	1.28 ± 0.24
	*P* value	0.24	0.03	0.43	0.26	0.78

**Table 3:** Dimensions of area parameters categorized by race.

	Race	C3	C4	C5	C6	C7
EPAu	Chinese	169.9 ± 39.2	183.5 ± 46.4	198.7 ± 59.5	234.1 ± 56.4	263.1 ± 64.8
	White	204.5 ± 42.4	221.4 ± 40.5	230.8 ± 39.6	264.2 ± 45.4	291.2 ± 47.2
	*P* value	0.00	0.00	0.02	0.03	0.06

EPAl	Chinese	203.1 ± 41.8	211.2 ± 49.9	237.6 ± 52.7	261.3 ± 59.1	281.5 ± 64.6
	White	231.2 ± 45.2	243.7 ± 50.1	266.1 ± 46.5	300.2 ± 51.8	316.8 ± 39.4
	*P* value	0.01	0.01	0.03	0.01	0.02

**Table 4:** Dimensions of linear parameters categorized by race and sex.

	Race and sex	C3	C4	C5	C6	C7
EPWu (mm)	Chinese female	14.6 ± 1.5	15.4 ± 1.7	15.8 ± 1.9	16.9 ± 1.7	18.8 ± 2.1
	White female	16.1 ± 1.8	16.9 ± 2.0	17.3 ± 1.5	18.4 ± 1.4	20.1 ± 1.8
	*P* value	0.02	0.03	0.03	0.01	0.08
	Chinese male	15.1 ± 2.1	16.2 ± 1.9	16.4 ± 1.6	17.6 ± 1.2	19.6 ± 1.4
	White male	17 ± 2.7	17.7 ± 1.1	17.9 ± 2.1	19.1 ± 1.4	20.9 ± 1.3
	*P* value	0.03	0.02	0.01	0.03	0.01

EPDu (mm)	Chinese female	13.0 ± 1.8	13.4 ± 1.8	13.4 ± 1.6	14.1 ± 1.7	15.4 ± 1.9
	White female	13.8 ± 1.5	14.5 ± 1.1	15.0 ± 1.6	16.4 ± 2.0	17.2 ± 1.9
	*P* value	0.30	0.06	0.00	0.00	0.01
	Chinese male	14.4 ± 1.9	14.9 ± 2.1	15.4 ± 2.33	15.9 ± 2.0	17.0 ± 1.9
	White male	14.8 ± 1.5	15.2 ± 2.1	15.4 ± 2.0	16.6 ± 2.2	18.0 ± 2.4
	*P* value	0.28	0.69	0.99	0.35	0.19

EPWl (mm)	Chinese female	15.1 ± 1.8	15.6 ± 1.9	16.7 ± 2.0	18.4 ± 2.7	20.3 ± 2.4
	White female	16.8 ± 1.9	17.4 ± 2.7	18.1 ± 1.4	19.1 ± 1.9	22.4 ± 2.9
	*P* value	0.02	0.03	0.04	0.44	0.08
	Chinese male	16.0 ± 2.0	16.5 ± 2.1	18.2 ± 2.3	20.2 ± 2.8	22.5 ± 3.0
	White male	18.0 ± 2.2	18.3 ± 2.8	20.1 ± 1.9	21.6 ± 2.4	23.2 ± 2.9
	*P* value	0.01	0.04	0.04	0.15	0.51

EPDl (mm)	Chinese female	14.9 ± 1.9	15.2 ± 1.9	15.3 ± 2.1	15.5 ± 2.0	15.8 ± 2.2
	White female	15.1 ± 2.2	15.6 ± 2.0	16.5 ± 2.4	17.6 ± 2.0	17.5 ± 2.0
	*P* value	0.78	0.12	0.01	0.01	0.03
	Chinese male	15.7 ± 1.7	16.0 ± 1.8	17.0 ± 2.1	17.3 ± 1.9	17.3 ± 2.2
	White male	16.1 ± 1.9	16.4 ± 2.3	17.1 ± 1.9	18.9 ± 1.5	18.4 ± 2.0
	*P* value	0.07	0.33	0.34	0.01	0.16

EPWu/EPDu	Chinese female	1.12 ± 0.16	1.16 ± 0.19	1.19 ± 0.18	1.20 ± 0.14	1.23 ± 0.16
	White female	1.17 ± 0.18	1.16 ± 0.06	1.16 ± 0.14	1.14 ± 0.18	1.19 ± 0.20
	*P* value	0.62	0.99	0.74	0.43	0.62
	Chinese male	1.08 ± 0.18	1.10 ± 0.17	1.11 ± 0.15	1.12 ± 0.14	1.16 ± 0.12
	White male	1.15 ± 0.15	1.18 ± 0.19	1.19 ± 0.20	1.17 ± 0.15	1.18 ± 0.19
	*P* value	0.26	0.22	0.20	0.22	0.86

EPWl/EPDl	Chinese female	1.02 ± 0.12	1.02 ± 0.11	1.1 ± 0.14	1.19 ± 0.12	1.30 ± 0.26
	White female	1.11 ± 0.11	1.11 ± 0.13	1.07 ± 0.20	1.09 ± 0.13	1.31 ± 0.28
	*P* value	0.04	0.04	0.59	0.03	0.99
	Chinese male	1.01 ± 0.14	1.02 ± 0.11	1.06 ± 0.14	1.19 ± 0.13	1.31 ± 0.19
	White male	1.08 ± 0.18	1.11 ± 0.12	1.17 ± 0.10	1.15 ± 0.17	1.27 ± 0.23
	*P* value	0.29	0.04	0.02	0.45	0.59

**Table 5:** Dimensions of area parameters categorized by race and sex.

	Race and sex	C3	C4	C5	C6	C7
EPAu	Chinese female	170.9 ± 33.2	180.6 ± 32.9	188.9 ± 32.5	214.5 ± 42.5	252.1 ± 47.1
	White female	199.2 ± 44.3	208.5 ± 42.5	224.4 ± 43.3	245.4 ± 51.2	278.2 ± 46.6
	*P* value	0.04	0.04	0.001	0.01	0.13
	Chinese male	171.2 ± 44.6	185.5 ± 57.6	207.1 ± 42.3	251.5 ± 33.7	274.5 ± 55.6
	White male	216.1 ± 49.6	228.8 ± 40.7	234.4 ± 41.2	275.4 ± 41.7	298.2 ± 50.6
	*P* value	0.01	0.03	0.04	0.03	0.23

EPAl	Chinese female	201.2 ± 42.1	203.6 ± 41.7	225.5 ± 48.7	246.1 ± 42.8	272.7 ± 49.8
	White female	202.7 ± 42.7	215.3 ± 43.1	245.1 ± 44.7	270.7 ± 47.8	285.2 ± 41.4
	*P* value	0.92	0.44	0.26	0.13	0.46
	Chinese male	204.6 ± 44.9	217.4 ± 57.6	248.2 ± 56.3	275.8 ± 60.3	290.7 ± 62.3
	White male	248.1 ± 42.7	260.3 ± 52.3	278.1 ± 54.4	317.7 ± 57.8	335.2 ± 36.4
	*P* value	0.01	0.04	0.14	0.03	0.03
